# Synergistic Effects of Injectable Platelet-Rich Fibrin and Bioactive Peptides on Dermal Fibroblast Viability and Extracellular Matrix Gene Expression: An In Vitro Study

**DOI:** 10.3390/molecules30163415

**Published:** 2025-08-19

**Authors:** Ana Giulia Lenci Paccola, Thomas Marcelino Couto dos Santos, Maria Clara Minelo, Thais Francini Garbieri, Mariana Liessa Rovis Sanches, Thiago José Dionísio, Rodrigo Cardoso de Oliveira, Carlos Ferreira Santos, Marília Afonso Rabelo Buzalaf

**Affiliations:** 1 Department of Biological Sciences, Bauru School of Dentistry, University of São Paulo, Bauru 17012-901, SP, Brazil; anagiulia@alumni.usp.br (A.G.L.P.); thomas.marcelino@usp.br (T.M.C.d.S.); mariaclaraminelo@usp.br (M.C.M.); tfgarbieri@usp.br (T.F.G.); thiagoj@usp.br (T.J.D.); rodrigocardoso@usp.br (R.C.d.O.); cfsantos@fob.usp.br (C.F.S.); 2 Hospital for Rehabilitation of Craniofacial Anomalies (HRAC), University of São Paulo, Bauru 17012-900, SP, Brazil; marianaanches@usp.br

**Keywords:** platelet-rich fibrin, peptides, fibroblasts, in vitro techniques, gene expression, collagen type I, hyaluronan synthases, fibronectins, rejuvenation, dermis

## Abstract

Facial aging is a multifactorial process involving changes in bone, fat compartments, ligaments, muscles, and skin. Collagen biostimulators, including synthetic agents and autologous platelet concentrates, have gained attention for facial rejuvenation. Injectable platelet-rich fibrin (i-PRF), a second-generation autologous concentrate, has shown promising regenerative properties due to its natural composition and growth factors. Cosmetic peptides, such as palmitoyl pentapeptide-4 (Matrixyl) and Tetrapeptide-21 (GEKG), are also studied for their ability to stimulate collagen synthesis and remodel the extracellular matrix. This in vitro study examined the potential synergistic effects of i-PRF combined with Matrixyl or GEKG on human dermal fibroblast viability, proliferation, and ECM-related gene expression. Fibroblasts were cultured under six conditions: control, i-PRF alone, Matrixyl alone, GEKG alone, i-PRF + Matrixyl, and i-PRF + GEKG. Viability and proliferation were assessed via MTT, crystal violet, and RealTime-Glo™ assays. Gene expression of *COL1A1*, *FN1*, and *HAS1* was measured using RT-qPCR. The combinations, especially i-PRF + GEKG, led to increased cell viability and upregulated ECM-related genes at 72 h. These effects were stronger than the individual treatments, suggesting synergistic effects, especially with GEKG. These findings highlight the clinical potential of combining autologous platelet concentrates with bioactive peptides for dermal regeneration. Further preclinical and clinical studies are warranted.

## 1. Introduction

Facial aging is a complex and multifactorial process involving changes across several anatomical compartments, including bone structure, fat distribution, facial ligaments, muscles, and skin. These alterations are the result of a combination of gravitational effects, bone resorption, tissue elasticity loss, and subcutaneous fat redistribution. To counteract these effects, a multimodal therapeutic approach is typically required, addressing key manifestations of aging, such as skin laxity, volume depletion, and surface damage [1,2]. Among various anti-aging interventions, collagen biostimulators play a crucial role by enhancing dermal thickness and improving skin texture and elasticity. Common synthetic biostimulators include calcium hydroxylapatite, poly-L-lactic acid, and polycaprolactone—materials recognized for their biocompatibility and biodegradability [3,4,5]. Despite their widespread use, these agents are synthetic and may carry inherent limitations in terms of cost, accessibility, or patient tolerance.

In contrast, autologous platelet concentrates—especially platelet-rich fibrin (PRF)—have emerged as potent natural alternatives [6]. These products offer a concentrated source of cytokines and growth factors with regenerative properties, obtained from the patient’s own blood, thereby minimizing immunogenic risks and adverse effects [6,7,8].

In facial aesthetics, two main types of autologous platelet concentrates have been used, namely, platelet-rich plasma (PRP) and platelet-rich fibrin (PRF) [6]. The former is obtained through double centrifugation using anticoagulants, which may impair healing by inhibiting the coagulation process, as clot formation following tissue injury is one of the initial steps in regeneration [8]. On the other hand, PRF is obtained without the use of anticoagulants, resulting in a three-dimensional matrix rich in growth factors that are released for an extended time, resulting in an enhanced regenerative potential. These platelet concentrates are derived from the patient’s own blood, thereby minimizing immunogenic risks and adverse effects [6,8]. Notably, injectable PRF (i-PRF), developed via low-speed centrifugation without anticoagulants, provides a fluid form of PRF that remains injectable for approximately 20 min before fibrin polymerization begins. This enables direct application for facial rejuvenation with promising outcomes in angiogenesis, fibroblast activation, and dermal regeneration [6,9,10,11]. More recently, horizontal centrifugation has been introduced as an alternative to traditional fixed-angle systems. In this method, tubes reach a 90° angle during rotation, which enhances density-based cell separation and reduces mechanical stress. Compared to fixed-angle centrifugation, the horizontal approach results in a more uniform layer separation, better preservation of cellular integrity, and higher platelet and leucocyte recovery, thereby improving the quality and consistency of i-PRF collection [12,13].

Simultaneously, the development of bioactive peptides has gained momentum in cosmetic dermatology. These peptides, composed of short chains of amino acids, can mimic natural extracellular matrix components and modulate gene expression linked to skin structure. Palmitoyl pentapeptide-4 (Matrixyl), derived from collagen type I sequences, stimulates fibroblast activity and promotes the production of collagen types I and III, as well as fibronectin [14]. Another peptide, Tetrapeptide-21 (GEKG), derived from collagen type IV sequences, is reported to induce greater upregulation of extracellular matrix genes, including *COL1A1*, *HAS1*, and *FN1*, making it a potentially more potent biostimulator than Matrixyl [15]. Despite individual studies supporting the regenerative properties of i-PRF and bioactive peptides, no previous study has explored their combined use. The current study addresses this gap by investigating whether the association between i-PRF and Matrixyl or GEKG results in synergistic effects on the viability, proliferation, and collagen-stimulating activity of primary human dermal fibroblasts in vitro.

## 2. Results

### 2.1. Crystal Violet Assay

**Figure 1:**
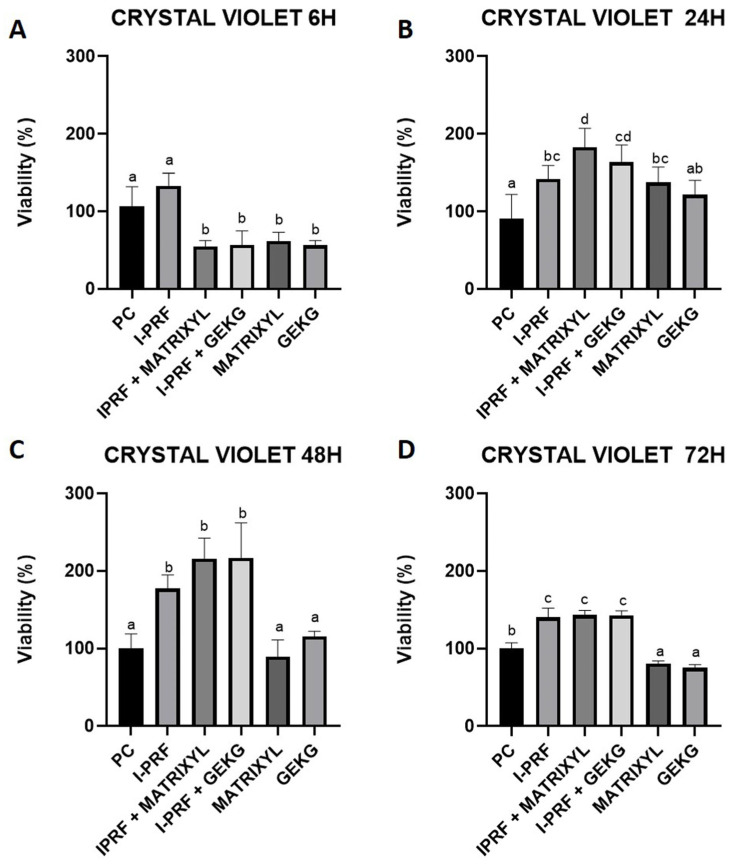
Cell viability based on absorbance in the crystal violet assay after treatment of FBH cells for (**A**) 6, (**B**) 24, (**C**) 48, or (**D**) 72 h. i-PRF was prepared by horizontal centrifugation at 300× *g* for 5 min. For each time point, distinct letters indicate significant differences between the groups (ANOVA and Tukey’s test, *p* < 0.05).

At 6 h (Figure 1A), all the treated groups, except for i-PRF alone, exhibited a significant decrease in % viability, when compared to the control and i-PRF (*p* < 0.05), which did not significantly differ from each other (*p* > 0.05). For the 24 h period (Figure 1B), GEKG alone did not significantly differ either from the control (*p* > 0.05) or from Matrixyl or i-PRF alone (*p* > 0.05). However, these treatments presented significantly increased % viability compared to the control (*p* < 0.05) but did not significantly differ from i-PRF + GEKG (*p* > 0.05). The highest increase in % viability was seen for i-PRF + Matrixyl, which differed from all the other groups, except for i-PRF + GEKG, which also presented increased % viability compared to the control. At 48 h (Figure 1C) and 72 h (Figure 1D), the % viabilities decreased for Matrixyl and GEKG alone compared to the 24 h results, making these groups similar to the control (*p* > 0.05). The highest % viabilities were found for i-PRF alone or associated with Matrixyl or GEKG, which did not significantly differ from each other (*p* > 0.05) but significantly differed from all the other groups (*p* < 0.05).

### 2.2. MTT Assay

**Figure 2:**
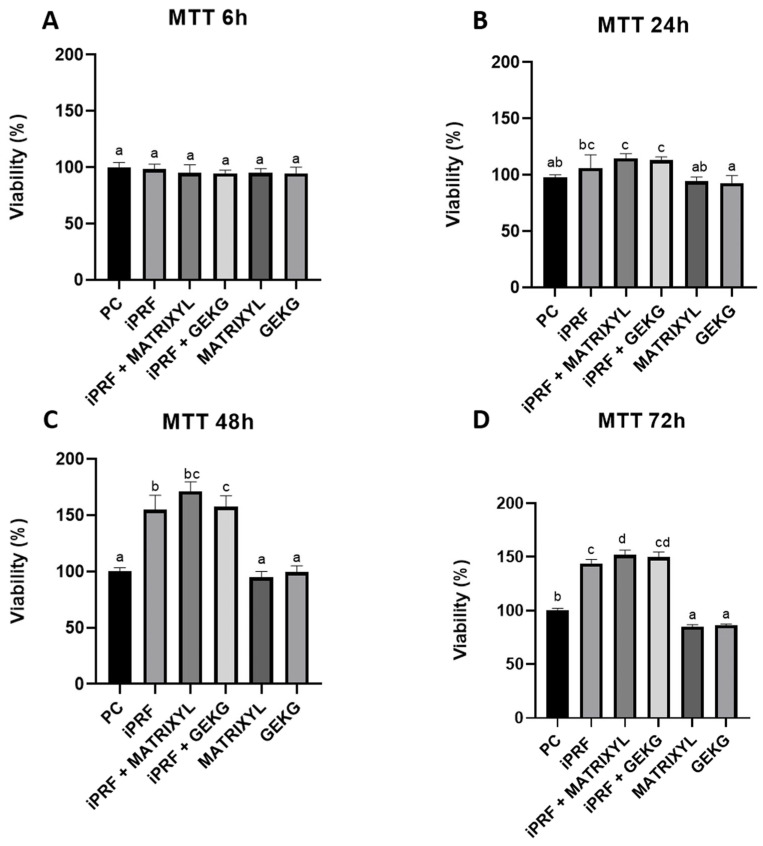
Cell viability based on absorbance in the MTT assay after treatment of FBH cells for (**A**) 6, (**B**) 24, (**C**) 48, or (**D**) 72 h. i-PRF was prepared by horizontal centrifugation at 300× *g* for 5 min. For each time point, distinct letters indicate significant differences between groups (ANOVA and Tukey’s test, *p* < 0.05).

At 6 h (Figure 2A), the metabolic activity was similar for all groups, with no significant differences among them (*p* > 0.05). At 24 h (Figure 2B), differences were seen between the groups. I-PRF alone or associated with Matryxil or GEGK presented increased % viabilities compared to the control, but the difference from the control was only significant for the associations (*p* < 0.05). On the other hand, the peptides alone showed slightly decreased % viabilities compared to the control, but the differences were not significant (*p* > 0.05). i-PRF alone did not significantly differ from i-PRF + Matryxil or i-PRF + GEKG (*p* > 0.05). At 48 (Figure 2C), an increase in % viability occurred for i-PRF alone and i-PRF associated with Matryxil or GEKG. These groups significantly differed from the control and from the groups treated with the peptides alone (*p* < 0.05). The latter two groups did not significantly differ either from each other or from the control (*p* > 0.05). The highest increase in % viability was seen for the association between i-PRF and Matryxil, which did not significantly differ either from i-PRF + GEKG or from i-PRF alone (*p* > 0.05). At 72 h (Figure 2D), all the groups treated with i-PRF (alone or combined) showed significantly greater viability than the control group (*p* < 0.05), with the highest viability observed in the i-PRF + Matrixyl group. The groups treated with the peptides alone presented the lowest viabilities, which were significantly reduced compared to the control (*p* < 0.05).

### 2.3. RealTime-Glo™ Cell Viability Assay

**Figure 3:**
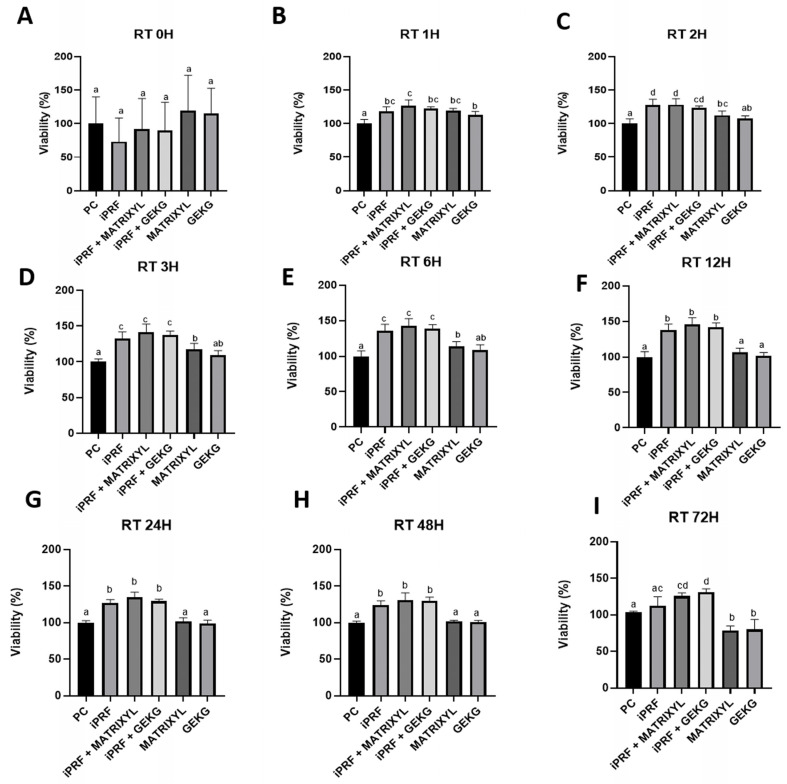
Cell viability based on luminescence in the RealTime-Glo™ assay after treatment of FBH cells for (**A**) 0, (**B**) 1, (**C**) 2, (**D**) 3, (**E**) 6, (**F**) 12, (**G**) 24, (**H**) 48, and (**I**) 72 h. i-PRF was prepared by horizontal centrifugation at 300× *g* for 5 min. For each time point, different letters indicate statistically significant differences between groups (ANOVA and Tukey’s test, *p* < 0.05).

At 0 h (Figure 3A), only the groups treated with the peptides alone showed slightly increased % viability when compared to the control. However, no significant differences between the groups were detected (*p* > 0.05). After 1 h (Figure 3B), all the treated groups exhibited significantly increased % viability compared to the control (*p* < 0.05). In addition, i-PRF + Matrixyl presented the highest % viability but differed significantly only from the GEKG alone and control groups (*p* < 0.05). At 2 h (Figure 3C), all the treated groups showed increased % viability compared with the control, but the difference was not significant for the GEKG group (*p* > 0.05). Matrixyl alone did not significantly differ from GEKG alone or i-PRF + GEKG (*p* > 0.05). The highest % viabilities were observed for i-PRF alone or in combination with Matrixyl, but this group did not significantly differ from i-PRF + GEKG (*p* > 0.05). At 3 h (Figure 3D) and 6 h (Figure 3E), a similar trend was observed; the only treated group that did not significantly differ from the control was GEKG alone (*p* > 0.05). Matrixyl did not significantly differ from GEKG. The highest % viabilities were found for the groups treated with i-PRF alone or in combination with the peptides, which did not significantly differ from each other (*p* > 0.05) but differed significantly from all the other groups (*p* < 0.05). At 12 h (Figure 3F), 24 h (Figure 3G), and 48 h (Figure 3H), the groups treated with the peptides alone did not significantly differ from each other or from the control (*p* > 0.05). The highest % viabilities were found for i-PRF alone or combined with the peptides, which did not significantly differ from each other (*p* > 0.05) but differed significantly from the control group and the groups treated with the peptides alone (*p* < 0.05). At 72 h (Figure 3I), the Matrixyl and GEKG alone groups did not significantly differ from each other (*p* > 0.05) but exhibited significantly reduced % viability when compared to the control (*p* < 0.05). i-PRF presented a slight increase in viability compared with the control, but the difference was not significant (*p* > 0.05). In addition, i-PRF alone did not significantly differ from i-PRF + Matrixyl. The highest % viabilities were found for the combinations of i-PRF with the peptides, which did not significantly differ from each other (*p* > 0.05) but showed significantly increased % viability compared to the control and the peptides alone (*p* < 0.05). i-PRF + Matrixyl did not significantly differ from i-PRF alone (*p* > 0.05). Supplementary Materials displays the same experimental data shown in Figure 3, rearranged as line graphs to highlight the temporal progression of the cell viability curves, without representing analyses of statistical significance.

### 2.4. RT-qPCR: Gene Expression of ECM Markers

Relative mRNA expression of extracellular matrix-related genes was assessed by RT-qPCR after 6, 24, and 72 h of treatment. Total RNA was isolated using a silica-membrane spin column system, and cDNA was synthesized using reverse transcriptase. Quantitative PCR was performed using TaqMan™ probes for *HAS1*, *COL1A1*, and *FN1*, normalized to *GAPDH* as a housekeeping gene. Expression levels were calculated using the 2^−ΔΔCt^ method.

The relative expression of the *COL1A1*, *FN1*, and *HAS1* genes was evaluated under different experimental conditions at 6, 24, and 72 h.

#### 2.4.1. *COL1A1*

**Figure 4:**
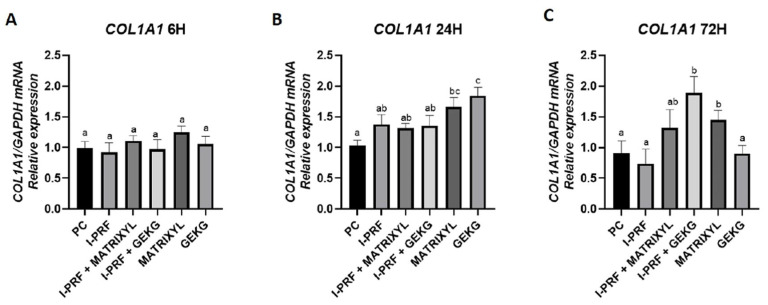
Relative mRNA expression of the *COL1A1* gene assessed by RT-qPCR after (**A**) 6, (**B**) 24, and (**C**) 72 h of treatment. For each time point, distinct letters indicate significant differences between groups (ANOVA and Tukey’s test, *p* < 0.05).

For *COL1A1* at 6 h (Figure 4A), no significant differences were observed among the groups (*p* > 0.05). However, at 24 h (Figure 4B), a significant increase in *COL1A1* expression was detected in the groups treated with Matrixyl and GEKG alone compared to the positive control (PC) group. The group treated with GEKG alone showed the highest relative expression (*p* < 0.05), which was significantly different from all the other treated groups, except for the Matrixyl alone group. A slight increase compared to the control was observed in the groups treated with i-PRF alone or in combination with the peptides, but these groups did not significantly differ either from the control or from the Matrixyl alone group. At 72 h (Figure 4C), the highest expression was observed for the group treated with iPRF + GEKG, which differed from all the other groups, except for the Matrixyl alone and i-PRF + Matrixyl groups (*p* < 0.05).

#### 2.4.2. *FN1*

**Figure 5:**
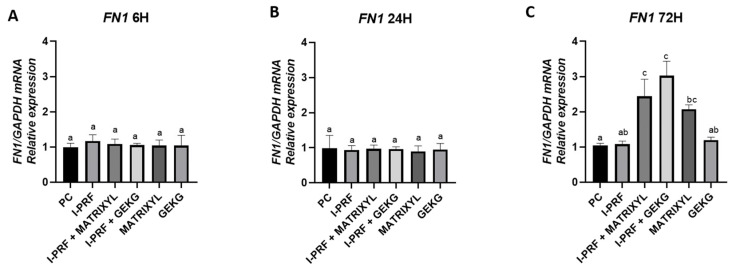
Relative mRNA expression of the *FN1* gene assessed by RT-qPCR after (**A**) 6, (**B**) 24, and (**C**) 72 h of treatment. For each time point, different letters indicate statistically significant differences between groups (ANOVA and Tukey’s test, *p* < 0.05).

No significant differences were observed among the groups at 6 h and 24 h (Figure 5A,B) (*p* > 0.05). However, at 72 h (Figure 5C), the groups treated with i-PRF combined with either Matrixyl or GEKG exhibited significantly increased *FN1* expression compared to the control (*p* < 0.05), suggesting a synergistic effect between i-PRF and the peptides at later time points. These groups, however, did not significantly differ from the Matrixyl alone group (*p* > 0.05), which also presented significantly increased FN1 expression at the 72 h time point compared to the control group (*p* < 0.05), but did not significantly differ from i-PRF or GEKG alone (*p* > 0.05).

#### 2.4.3. *HAS1*

**Figure 6:**
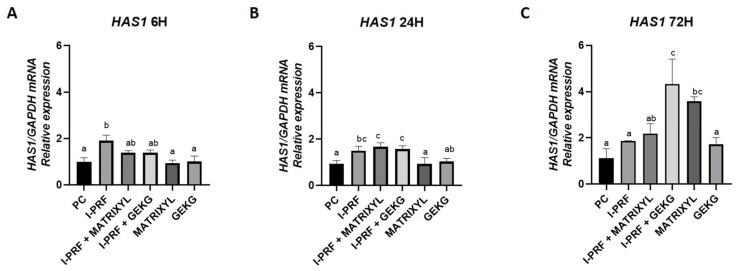
Relative mRNA expression of the *HAS1* gene assessed by RT-qPCR after (**A**) 6, (**B**) 24, and (**C**) 72 h of treatment. Different letters indicate statistically significant differences between groups (*p* < 0.05).

The only treatment that promoted a significant increase in expression at 6 h (Figure 6A) compared with the control group was the one with i-PRF alone (*p* < 0.05). However, this group did not significantly differ (*p* > 0.05) from the groups treated with the combinations (i-PRF + Matrixyl and i-PRF + GEKG). At 24 h (Figure 6B), a more pronounced upregulation was observed in the groups treated with i-PRF + Matrixyl and i-PRF + GEKG, both showing significantly higher expression levels than the control (*p* < 0.05). However, these groups did not significantly differ from the i-PRF alone group, which still presented significantly higher expression levels compared to the control (*p* < 0.05). At 72 h (Figure 6C), the i-PRF + GEKG group exhibited the highest *HAS1* expression, differing from all the groups, except for Matrixyl alone (*p* < 0.05), which also presented significantly higher expression compared to the control (*p* < 0.05) but did not significantly differ from i-PRF + Matrixyl (*p* > 0.05). i-PRF alone, i-PRF + Matrixyl, and GEKG alone did not significantly differ from the control (*p* > 0.05).

## 3. Discussion

This study explored the in vitro effects of injectable platelet-rich fibrin (i-PRF), both alone and in combination with two bioactive peptides—Matrixyl and Tetrapeptide-21 (GEKG)—on human dermal fibroblasts. The findings demonstrate a potential synergistic effect between i-PRF and the peptides, especially GEKG, in enhancing cell viability and extracellular matrix (ECM)-related gene expression, suggesting promise for aesthetic and regenerative applications.

Regarding cell viability, we employed three distinct assays that provided complementary information on the cell behavior in function of the different treatments. They revealed distinct cell responses across the treatments and time points. At early stages (6 h), the MTT assay did not detect significant differences between the groups, suggesting limited short-term metabolic impact. However, the crystal violet assay, which quantifies adherent cells based on DNA and protein content, revealed decreased viability in the groups treated with peptides alone or in combination, indicating early anti-adhesive effects of the peptides. Notably, i-PRF alone maintained comparable viability to the control. These results align with previous studies highlighting i-PRF’s safety profile and regenerative properties as an autologous agent rich in growth factors and cytokines [7,9,11,16].

Over longer periods (24–72 h), the divergence between the treatments became more pronounced. The peptides alone consistently tended to reduce cell viability relative to the control, indicating possible cytotoxic effects, particularly at 72 h, while i-PRF, either alone or in combination, significantly enhanced cell viability. The combinations tended to produce greater effects than the isolated components, supporting a synergistic mechanism. Interestingly, the crystal violet assay remained more sensitive than MTT throughout the timeline, likely due to its ability to detect changes in cell number and adherence, reflecting fibroblast behavior more accurately under certain stimuli [17].

The RealTime-Glo™ Cell Viability Assay further confirmed dynamic changes in metabolic activity. An increase in luminescence relative to the control group was observed in the early hours (1–6 h), more pronounced for the i-PRF-treated groups, indicating a rapid cellular response. Over time (12–48 h), the differences between the groups became more pronounced, with those treated with i-PRF (alone or in combination) presenting significantly increased luminescence compared to the control, while the groups treated with the peptides alone did not significantly differ from the control. At 72 h, however, the only groups that presented significantly higher luminescence compared to the control were those treated with the combination of i-PRF and the peptides, while the groups treated with the peptides alone showed significantly reduced luminescence compared to the control, reinforcing the potential synergistic action between i-PRF and the peptides.

RT-qPCR was performed to evaluate the mRNA levels of *COL1A1*, *FN1*, and *HAS1*, in terms of ECM gene expression. These genes are critical for collagen production, fibronectin-mediated cell adhesion, and hyaluronic acid synthesis, respectively—key elements in skin structure and regeneration [18]. Regarding *COL1A1*, the main skin collagen, none of the treatments increased its expression at 6 h (Figure 4A). At 24 h (Figure 4B), there was a significant increase in expression with respect to the control upon treatment with GEKG alone (1.7-fold) and Matrixyl alone (1.5-fold). This result is in line with the findings of Fairwick et al. [18], although those authors found an increase of 2.8-fold and 1.8-fold in *COL1A1* expression after treatment with GEKG and KTTKS (similar to Matrixyl), respectively. However, in their study, the fibroblasts were derived from forehead skin (as opposed to the abdomen in the present study), and the concentration of GEKG employed was 1 ppm (versus 10 ppm in the present study). At 72 h (Figure 4C), while *COL1A1* expression remained the same in the group treated with Matrixyl alone, the group treated with GEKG alone showed a substantial decrease in *COL1A1* expression, reaching levels similar to the control group. The highest *COL1A1* expression at this time point was observed for the combination i-PRF + GEKG, which was nearly 2-fold higher than that of the control. These findings once again reinforce the synergistic action of the combination i-PRF + GEKG.

Regarding *FN1* expression, related to cell adhesion, at 6 h (Figure 5A) and 24 h (Figure 5B), none of the groups showed increased expression compared to the control group. These results contrast with the study by Fairwick et al. [18], which reported an increase in *FN1* expression by nearly 20-fold and 1.5-fold after treatment with GEKG and KTTSS, respectively. Again, it should be noted that Fairwick et al. employed fibroblasts from a different location [18] and used a 10-fold lower concentration of GEKG (1 ppm). After 72 h (Figure 5C), however, the combinations of i-PRF + GEKG and i-PRF + Matrixyl significantly increased *FN1* expression by 3.0-fold and 2.5-fold, respectively, compared to the control. Matrixyl alone also resulted in significantly increased *FN1* expression (around 2-fold) compared to the control. It should be highlighted that the groups treated with the peptides alone had significantly reduced cell viability at 72 h, as evaluated by the crystal violet (Figure 1D) and MTT assays (Figure 2D). Thus, the reduced cell numbers might have impacted the lower expression of FN1 observed in the present study. The association between i-PRF and the peptides appears to counteract this effect.

Regarding *HAS1*, one of the hyaluronan-synthesizing enzymes, i-PRF alone increased its expression by around 2-fold compared to the control in the short term (6 h), with expression levels remaining relatively stable over 72 h (Figure 6C). At 24 h (Figure 6B), the groups treated with i-PRF alone or combined with the peptides had a significant increase (around 1.8-fold) in *HAS1* expression relative to the control, while the groups treated with the peptides alone did not differ from the control. Over time (72 h), although the groups treated with i-PRF alone or i-PRF associated with Matrixyl showed a near 2-fold increase in *HAS1* expression compared with the control, the highest expression levels were found for the combination of i-PRF + GEKG and Matrixyl alone (around 4.0-fold and 3.5-fold relative to the control, respectively). GEKG alone, regardless of the evaluation period, was not able to increase HAS1 expression. Fairwick et al. [18] reported increased HAS1 expression of 10.0-fold and 5.0-fold after treatment of human primary forehead fibroblasts with 1 ppm Matrixyl or GEKG, respectively, for 24 h.

Our RT-qPCR results revealed that the most significant differences were observed at 72 h. The i-PRF + GEKG-treated group exhibited the most consistent upregulation across markers, suggesting a particularly potent combination. However, when GEKG was employed alone, this upregulating effect was not observed. When these results are analyzed in conjunction with viability test outcomes (Figure 1, Figure 2 and Figure 3), it becomes evident that at later time points (48 and 72 h), the peptides alone led to significantly reduced cell viability compared to the control. This might explain their lack of effect on ECM-related gene expression. Conversely, the groups treated with i-PRF alone or in combination with the peptides consistently showed higher cell viability compared to the control from 24 to 72 h. Thus, the higher cell number might be related to the increased upregulation of ECM markers. In the study by Fairwick et al. [18], ECM gene expression was evaluated after treatment of primary human forehead fibroblasts for 24 h. The authors observed that GEKG alone was a more potent stimulator of these genes than KTTKS (Matrixyl). However, longer periods were not evaluated in that study. In the present study, Matrixyl alone was able to increase mRNA expression for all genes tested at 72 h (Figure 4C, Figure 5C and Figure 6C), despite showing a reduction in cell viability at the same time points in the viability tests (Figure 1, Figure 2 and Figure 3). However, the expression of ECM-related genes promoted by Matrixyl was lower than that observed for the combination of i-PRF + GEKG, which demonstrated the best performance among all the treatments evaluated.

Although the present study did not investigate the underlying molecular mechanisms, it is plausible to speculate that the combination of i-PRF and bioactive peptides promoted the simultaneous activation of multiple intracellular signaling pathways related to fibroblast proliferation and extracellular matrix synthesis. Growth factors present in i-PRF, such as platelet-derived growth factor (PDGF), transforming growth factor-beta (TGF-β), and vascular endothelial growth factor (VEGF), are known to activate pathways including PI3K/Akt, MAPK/ERK, and TGF-β/Smad, all of which are involved in fibroblast proliferation and the production of collagen and hyaluronic acid [19,20,21]. On the other hand, bioactive peptides, such as Matrixyl (palmitoyl pentapeptide) and Tetrapeptide-21 (GEKG), have been associated with the upregulation of ECM-related genes through mechanisms that include modulation of gene expression via cell surface receptors and activation of transcription factors, such as AP-1 and Smad proteins [18,22]. The possible convergence or synergistic interaction between signaling pathways activated by i-PRF and those induced by the peptides may explain the observed increase in *COL1A1*, *FN1*, and *HAS1* gene expression in the combination groups, especially after 72 h of exposure.

GEKG was first reported in studies using in silico approaches aimed at identifying highly repetitive amino acid motifs in several ECM proteins [23,24]. It is now a cosmeceutical peptide commercially available in the product TEGO^®^ Pep 4-17 (Evonik Industries, Essen, Germany). However, only a few studies evaluated its potential for skin rejuvenation. Fairwick et al. [18] evaluated the efficacy of GEKG in vivo. Ten volunteers (>35 years) were treated in a double-blind, randomized, placebo-controlled study once a day for 8 weeks with either vehicle only (placebo) or the same vehicle containing 50 ppm GEKG. Expression of *COL1A1* (in biopsies of buttock skin) was significantly increased by GEKG compared with the placebo. Additionally, histochemical analyses showed that the treatment with GEKG increased the formation of procollagen, hyaluronic acid, and fibronectin. Furthermore, the penetration of GEKG into the skin was shown to increase ex vivo when nano-sized carrier systems (emulsion w/o) were employed, instead of standard creams [18]. The present study is the first to evaluate the combination of GEKG with i-PRF. Our results revealed that i-PRF can mitigate the potential inhibitory effects of GEKG on cell viability while simultaneously enhancing ECM gene-related outcomes. The autologous origin of i-PRF provides excellent biocompatibility and immunological safety [6,7], reducing risks associated with synthetic materials. This study has some limitations that should be acknowledged. First, the experiments were conducted exclusively in vitro using human dermal fibroblasts derived from abdominal skin. It is well established that fibroblasts from different anatomical sites display distinct biological behaviors and gene expression profiles, particularly when comparing sun-protected areas, such as the abdomen, with photoexposed regions, like the face [25]. Second, the use of monolayer cultures may not fully replicate the complex three-dimensional architecture and cellular interactions found in native skin tissue [26]. Furthermore, this study evaluated only a single concentration for each peptide and did not explore dose–response relationships or longer follow-up periods beyond 72 h. Future studies should aim to validate these findings in three-dimensional skin models and in vivo systems, which would allow for a more comprehensive assessment of tissue remodeling, neocollagenesis, and long-term biocompatibility. Additionally, mechanistic studies investigating the specific intracellular signaling pathways activated by the combination of i-PRF and bioactive peptides would provide valuable insights. Clinical trials focusing on safety, efficacy, and patient-reported outcomes will be essential before translating these findings into routine aesthetic or regenerative practice. Furthermore, this study used PRF from a single donor to ensure experimental consistency across assays. Since the composition of growth factors in PRF exhibits interindividual variability, the results may reflect the specific profile of that donor. Future studies will include samples from multiple donors and/or a standardized pool to capture the average response and estimate biological variability.

In addition, the present study focused on i-PRF, a liquid material that is fully resorbed within a typical 2–3-week period. In the last few years, a novel heating process has been shown to extend the working properties of PRP/PRF toward a duration of 4–6 months. The resulting material is known as extended-PRF (e-PRF) or Albumin-PRF (Alb-PRF) and has been employed in facial aesthetics as a natural filler (biofiller). This material has many advantages over i-PRF, such as enhanced structural integrity, slower degradation, and sustained growth factor release [27]. Thus, evaluating the potential synergistic effect between Alb-PRF and bioactive peptides in facial aesthetics becomes particularly interesting.

## 4. Materials and Methods

### 4.1. Ethical Approval

This study was approved by the Research Ethics Committee of the Faculty of Dentistry of Bauru, University of São Paulo (FOB-USP), under protocol CAAE 78245523.4.0000.5417, in accordance with Brazilian Resolution CNS 466/12. Blood collection from healthy volunteers was conducted after obtaining written informed consent.

### 4.2. Cell Culture

Primary human dermal fibroblasts (FBH CK 001) were obtained from skin samples (abdominoplasty) provided by the Kosmoscience Group (Valinhos, Brazil). The cells were cultured in Dulbecco’s Modified Eagle Medium/Nutrient Mixture F-12 (DMEM/F-12) medium supplemented with 10% fetal bovine serum (FBS) and 1% antibiotic solution, maintained at 37 °C in a 5% CO_2_ humidified atmosphere.

### 4.3. Preparation of Biostimulatory Agents

Injectable platelet-rich fibrin (i-PRF) was prepared using blood collected from three healthy adult volunteers (FOB-USP, Bauru, Brazil), following strict inclusion/exclusion criteria and with ethical approval. The inclusion criteria were as follows: age between 26 and 41 years, no use of any medication, and an unremarkable medical history. Volunteers not meeting the inclusion criteria or presenting hematological, cardiac, renal, pulmonary, hepatic, or autoimmune diseases; diabetes; hyperthyroidism; leprosy; tuberculosis; cancer; abnormal bleeding; seizures; or infectious transmissible diseases, such as Chagas disease, hepatitis, AIDS, or syphilis, were excluded. Whole blood was drawn into 9 mL plastic tubes without anticoagulant (Vacuette tube, code 455001BR, Greiner Bio-One, Americana, SP, Brazil) and centrifuged horizontally at a maximum relative centrifugal force (RFC max) of 300 g for 5 min (BIOPRF, Fort Lauderdale, FL, USA) [28,29,30]. The plasma layer above the red blood cells was carefully aspirated (approx. 1 mL per tube) and stored on ice until use. A conditioned medium was produced by incubating 1 mL of i-PRF with 5 mL of DMEM/F-12 without fetal bovine serum (FBS) in 6-well plates for 72 h at 37 °C, with agitation every 12 h. The supernatant (approximately 6 mL) was collected and used at a ratio of 20% i-PRF conditioned medium and 80% complete culture medium for the treatments [28].

Matrixyl 3000 (CAS #214047-00-4) and Tetrapeptide-21 (GEKG) (CAS #960608-17-7) were purchased from RS Synthesis (Louisville, KY, USA). Stock solutions (500 ppm and 100 ppm) were prepared in Milli-Q water and filtered. Final working concentrations were set at 10 ppm for all experiments [18].

### 4.4. Experimental Design and Cell Culture

Primary human dermal fibroblasts were cultured in DMEM/F-12 supplemented with 10% FBS and 1% penicillin/streptomycin and maintained at 37 °C in a humidified 5% CO_2_ atmosphere. The cells were exposed to six treatment groups, as follows:

- PC: positive control (DMEM + 10% FBS + 1% penicillin/streptomycin);
- G1: i-PRF;
- G2: i-PRF + Matrixyl (10 ppm);
- G3: i-PRF + GEKG (10 ppm);
- G4: Matrixyl (10 ppm);
- G5: GEKG (10 ppm).

### 4.5. Cell Viability and Proliferation

Cell viability, proliferation, toxicity, and metabolic activity were assessed using MTT, crystal violet, and RealTime-Glo™ assays at multiple time points.

For the crystal violet assay, cells were seeded at a density of 1 × 10^4^ in 96-well plates containing 200 µL of culture medium. Adhesion was allowed for 24 h. After this period, the culture medium was removed, and 200 µL of each treatment was added. After the experimental periods (6, 24, 48, and 72 h), the cells were fixed and stained with 0.05% crystal violet solution for 20 min. Excess dye was removed, and the remaining stain was solubilized in methanol [17]. Absorbance (FLUOstar OPTIMA, BMG Labtech, Offenburg, Germany) was measured at 570 nm [31,32]. The absorbance of each reaction was converted to cell viability (%) using the following equation: (absorbance treatment × 100)/absorbance control [33]. Crystal violet stains the nucleic acids of viable adherent cells.

Cellular metabolic activity was assessed using the MTT assay at 6, 24, 48, and 72 h. Cells (1 × 10^4^) were incubated with 0.5 mg/mL MTT solution for 4 h. After this period, the MTT solution was removed, and the cells were resuspended in 200 µL of dimethylsulfoxide (DMSO). The resulting formazan crystals were dissolved in DMSO. Absorbance was measured at 550 nm (FLUOstar OPTIMA, BMG Labtech, Offenburg, Germany) [33,34,35] and converted into % cell viability.

The RealTime-Glo™ MT Cell Viability Assay (PROMEGA, Madison, WI, USA) was used to continuously monitor metabolic activity. Cells were seeded (1 × 10^4^) in 96-well plates containing 200 µL of culture medium. The assay reagent was added to each well, and luminescence (FLUOstar OPTIMA, BMG Labtech, Offenburg, Germany) was measured at multiple time points over 72 h (0, 1, 2, 3, 6, 12, 24, 48, and 72 h), without removing or lysing the cells.

### 4.6. Gene Expression Assays

Gene expression of extracellular matrix markers [type 1A1 collagen (*COL1A1*), 1A hyaluronic acid (*HAS1*), and fibronectin (*FN1*)] was quantified via RT-qPCR using standard protocols, with *GAPDH* as the housekeeping gene.

Total RNA was obtained directly from cells using the PureLink RNA Mini Kit (Invitrogen, Carlsbad, CA, USA) according to the manufacturer’s instructions. The spectrophotometer NanoDrop^TM^ 1000 (Thermo Fisher Scientific, Waltham, MA, USA) was used for RNA concentration measurement and quality assessment. cDNA was synthesized using a High-Capacity cDNA Reverse Transcription Kit (Applied Biosystems, Foster City, CA, USA). Real-time quantitative polymerase chain reaction (RT-qPCR) was performed using a gene expression assay and proprietary primers with Taqman^TM^ Gene Expression PCR Master Mix (Applied Biosystems^TM^) targeting mRNA for *HAS1* (Hs04398914_m1), *FN1* (Hs01549976_m1), and *COL1A1* (Hs0016004_m1). *GAPDH* (Hs99999905_m1) was used as a reference gene. All experiments were performed in the ViiA^TM^ 7 Real-Time PCR System (Applied Biosystems^TM^) using the comparative cycle threshold (Ct) method (∆∆Ct), as previously described [35].

### 4.7. Statistical Analysis

The software GraphPad Prism, version 8.2 (GraphPad Software Inc., La Jolla, CA, USA) was used. Initially, normality (Kolmogorov–Smirnov test) and homogeneity (Bartlett’s test) were checked. With these criteria satisfied, the data were tested by Analysis of Variance (ANOVA) and Tukey’s post hoc test, with a significance level of 5%.

## 5. Conclusions

The findings of this study support the synergistic benefits of combining injectable platelet-rich fibrin (i-PRF) with bioactive peptides, especially GEKG, in promoting dermal fibroblast viability and stimulating ECM gene expression. The association between i-PRF and GEKG enhanced cell activity and collagen production more effectively than either component alone, particularly at later time points. These results underscore the therapeutic potential of combining autologous regenerative biomaterials with targeted peptides to improve skin quality and support tissue regeneration. Further in vivo studies are warranted to validate these outcomes and establish their relevance in clinical settings focused on facial rejuvenation and regenerative dermatology.

## Data Availability

The data presented in this study are available upon request from the corresponding authors.

## Supplementary Materials

The following supporting information can be downloaded at https://www.mdpi.com/article/10.3390/molecules30163415/s1.

## Abbreviations

The following abbreviations are used in this manuscript:

I-PRF	Injectable Platelet-Rich Fibrin
GEKG	Tetrapeptide-21
COL1A1	Collagen Type I Alpha-1
FN1	Fibronectin 1
HAS 1	Hyaluronan Synthase 1
PRF	Platelet-Rich Fibrin
FBH	Human Dermal Fibroblasts
ECM	Extracellular Matrix
PDGF	Platelet-derived Growth Factor
TGF-	Transforming Growth Factor-beta
VEGF	Vascular Endothelial Growth Factor
FBS	Fetal Bovine Serum
DMEM/F-12	Dulbecco’s Modified Eagle Medium/Nutrient Mixture F-12
DMSO	Dimethylsulfoxide
FBH CK 001	Primary Human Dermal Fibroblasts
