# Supplementary figures and images for: Synergistic Effects of Injectable Platelet-Rich Fibrin and Bioactive Peptides on Dermal Fibroblast Viability and Extracellular Matrix Gene Expression: An In Vitro Study
Source: Molecules. 2025 Aug 19;30(16):3415. doi: 10.3390/molecules30163415 (PMC12388378; doi:10.3390/molecules30163415)

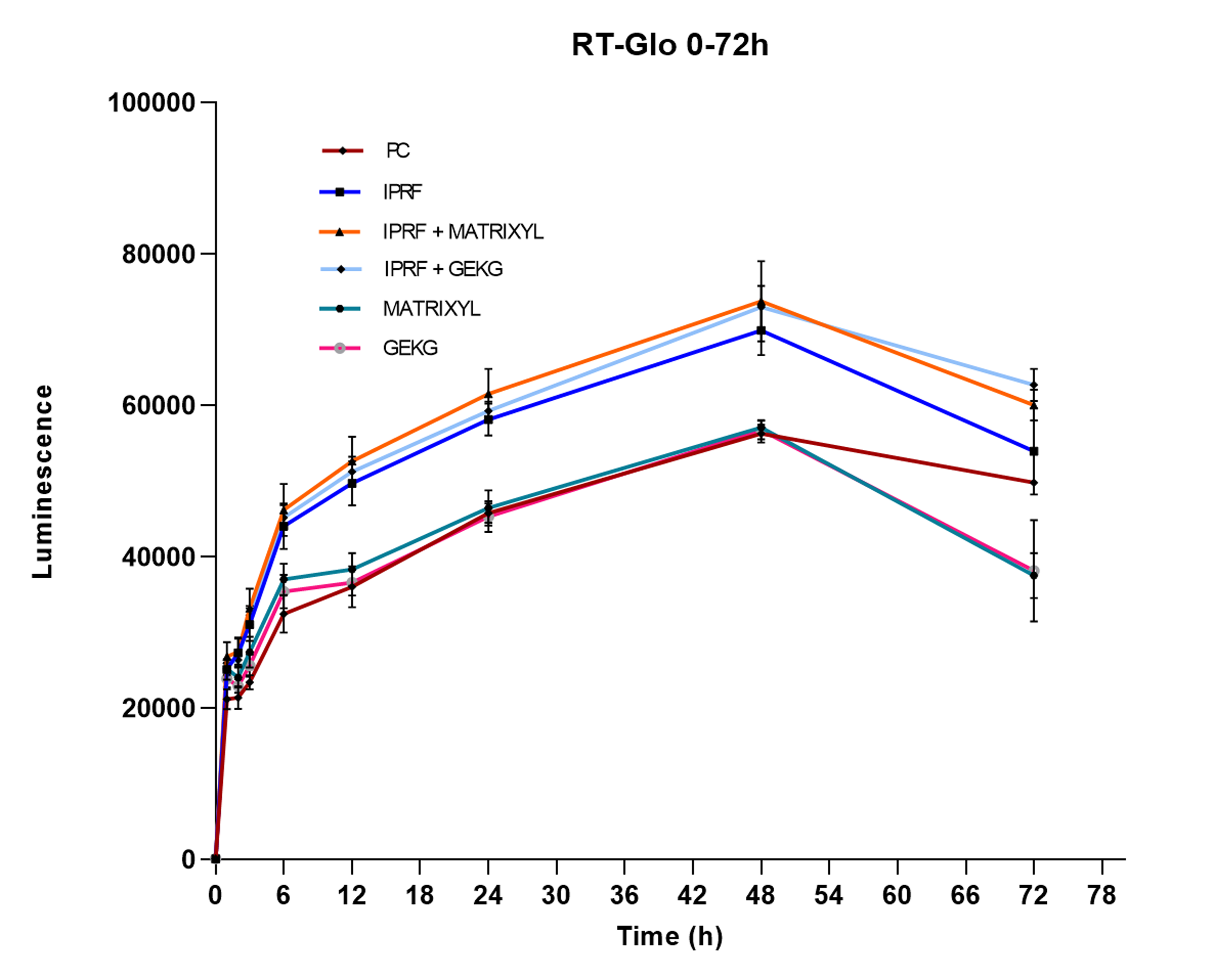

Supplement: Supplementary file 1 [file molecules-30-03415-s001.zip › molecules-3776827-supplementary.tif]
